# Maternal diabetes and the role of neonatal reticulocyte hemoglobin content as a biomarker of iron status in the perinatal period

**DOI:** 10.3389/fendo.2022.1011897

**Published:** 2022-11-08

**Authors:** Evgeniya Babacheva, Dimitrios Rallis, Helen Christou, George Mitsiakos, Themistoklis Mikos, Kalliopi Dampala, Christos Tsakalidis, Anna Kioumi, Dimitrios G. Goulis, Vasiliki Soubasi

**Affiliations:** ^1^ Second Department of Neonatology, Aristotle University of Thessaloniki, School of Medicine, Thessaloniki, Greece; ^2^ Brigham and Women’s Hospital, Harvard Medical School, Boston, MA, United States; ^3^ Unit of Reproductive Endocrinology, First Department of Obstetrics and Gynecology, Aristotle University of Thessaloniki, School of Medicine, Thessaloniki, Greece; ^4^ Department of Hematology, Aristotle University of Thessaloniki, School of Medicine, Thessaloniki, Greece

**Keywords:** anemia, gestational diabetes, hypochromia, iron deficiency, reticulocytes

## Abstract

**Aims:**

We aimed to evaluate the effects of maternal diabetes on neonatal iron status, measuring erythrocyte indices including hemoglobin, hematocrit, reticulocytes, mean corpuscular volume (MCV), percent (%) hypochromia, ferritin, and additionally mean reticulocyte hemoglobin content (MCHr) as an early marker of iron deficiency, and examine the association between neonatal MCHr, red cell indices, and ferritin.

**Materials and Methods:**

We conducted a hospital-based prospective cohort study in a tertiary neonatal unit of a University Hospital from 2018 to 2020. We enrolled 126 maternal-infant pairs of mothers whose pregnancy was associated with diabetes and 74 maternal-infant pairs from uncomplicated pregnancies. Erythrocyte indices were analyzed within the first twelve hours after birth. Erythrocyte parameters were compared between infants of the diabetes and the non-diabetic group. We examined the correlation of the neonatal MCHr with perinatal characteristics, including gestation, birth weight, maternal body mass index, the erythrocytic indices, maternal diabetes, maternal obesity, prematurity, small-for-gestational-age status, maternal preeclampsia, and maternal anemia. Finally, we evaluated the discordance between neonatal MCHr and neonatal ferritin.

**Results:**

Infants of the diabetes group had a significantly lower MCHr (32.6 pg vs. 34.2 pg, p=0.003) compared with infants of uncomplicated pregnancies. Neonatal MCHr was significantly correlated with maternal hypochromia (r=-0.237, p=0.004) and neonatal MCV (r=0.674, p<0.001). Neonatal MCHr was significantly associated with maternal diabetes [standardized coefficients 0.21, 95% confidence interval (CI) 0.05-0.58, p=0.003) and maternal preeclampsia (standardized coefficients 0.17, 95% CI 0.02-0.92, p=0.019), after adjusting for maternal anemia, maternal obesity, prematurity, and small-for-gestational-age status. Those results were consistent also when analyzing maternal-infant pairs with pre-existing diabetes, and maternal-infant pairs with gestational diabetes. There was significant discordance between neonatal MCHr and neonatal ferritin (p=0.001).

**Conclusions:**

MCHr was significantly lower in infants of mothers whose pregnancy was associated with diabetes compared with infants of non-diabetic mothers and correlated with neonatal and maternal red cell indices of iron deficiency. Since there was significant discordance between neonatal MCHr and ferritin during the first postnatal day, it is possible that MCHr could be used as a screening test for iron deficiency, especially in infants.

## Introduction

Iron forms an important co-factor of enzymes involved in cell replication, myelination, neurotransmitter synthesis, and cellular energy metabolism, playing a key role in brain development (1, 2). Several factors influence iron status at birth, including maternal nutrition and morbidities. Prematurity impacts iron stores since these are mainly accrued during the third trimester of pregnancy (3). Also, maternal diabetes mellitus is associated with depleted fetal iron stores, and this is proportionate to the degree of maternal glycemic control (4, 5). Previous research has described the impact of altered glucose handling *in utero* on fetal iron deficiency through chronic hypoxia and increased erythropoiesis (6). Current evidence has suggested that infants of mothers whose pregnancy was complicated by diabetes (infants of diabetic mothers, IDM) are at increased risk for developing iron deficiency resulting in neurocognitive impairment later in life (4–6).

Of note, iron deficiency, even before the manifestation of anemia, may contribute to impaired psychomotor development with potential permanent deficits (1, 7, 8); therefore, early recognition of iron deficiency in high-risk individuals is essential. Iron deficiency is characterized by microcytic, hypochromic erythrocytes and low iron stores. Microcytosis is revealed by the low mean corpuscular volume (MCV), which measures the average red blood cell volume, whereas hypochromia by a low mean corpuscular hemoglobin concentration, which is the measure of the concentration of hemoglobin in a given volume of packed red blood cells (9). Previous studies have suggested that ferritin, soluble transferrin receptor, transferrin saturation, and zinc protoporphyrin were poor predictors of iron deficiency in children (9). The ferritin concentrations may be misleading in the presence of acute or chronic inflammation as ferritin is also an acute phase reactant. In contrast, mean reticulocyte hemoglobin content (MCHr), which reflects the availability of iron for bone marrow hemoglobin production during the prior 24-48 h, has been proposed as a tool for evaluating the iron status and a sensitive predictor for later anemia (10). Previous studies have evaluated MCHr concentrations in term and preterm infants, suggesting that MCHr indicates iron deficiency with a better consistency compared with other indices, such as ferritin (3, 10, 11); however, evidence regarding the impact of maternal diabetes on those specific erythrocytic parameters is limited.

Given the significance of the early detection of iron deficiency in high-risk neonates such as the IDMs for the long-term neurodevelopment, this study aimed to examine the effects of maternal diabetes on neonatal iron status after birth, measuring erythrocyte indices including hemoglobin, hematocrit, reticulocytes, MCV, percent (%) hypochromia, and additionally neonatal MCHr. Moreover, the study aimed to examine the possible discordance between neonatal MCHr and neonatal ferritin among IDMs and infants from uncomplicated pregnancies.

## Materials and methods

### Study population

**Figure 1:**
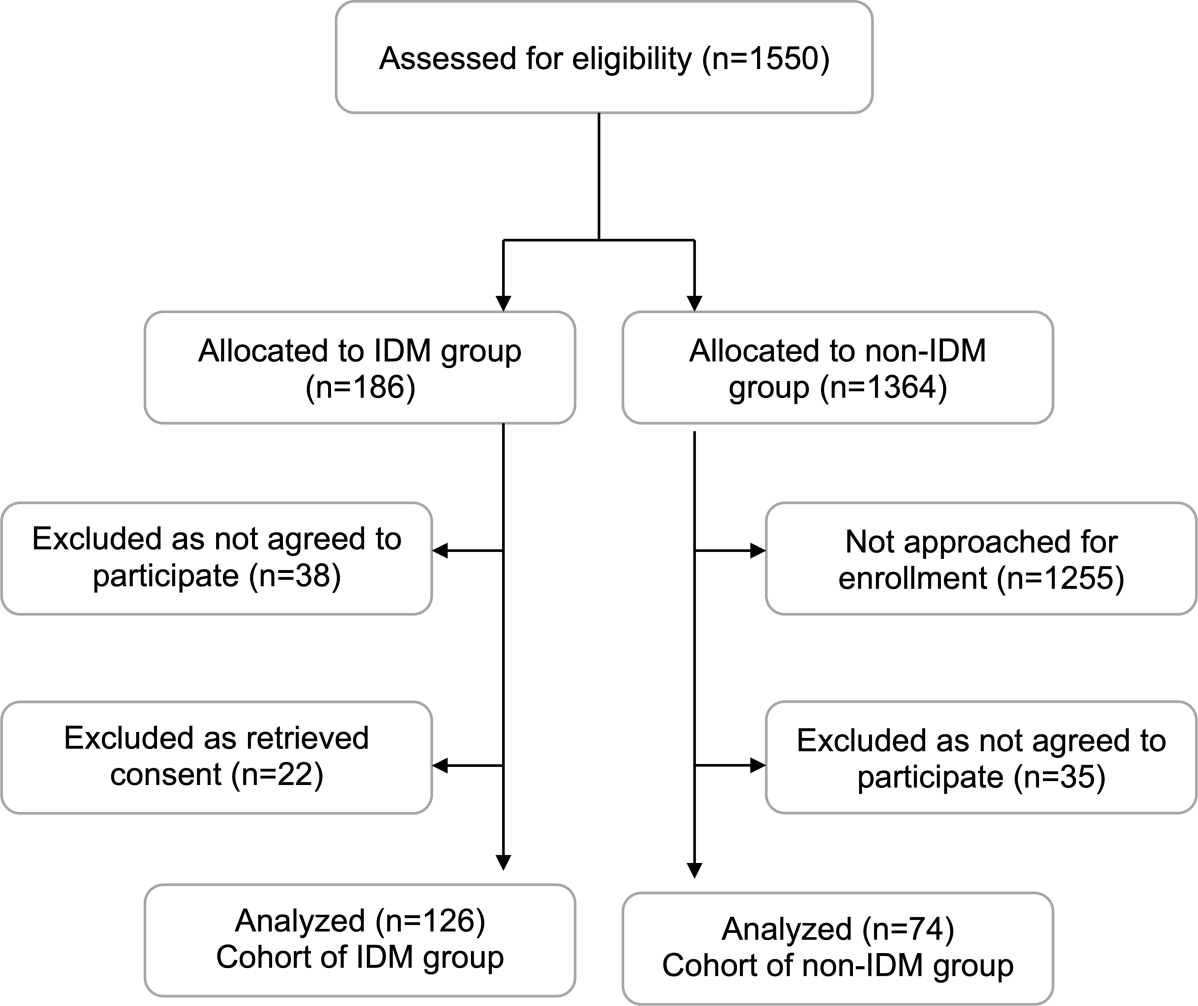
Flowchart of the study population. IDM, infant of diabetic mother.

We conducted a hospital-based prospective cohort study in a tertiary neonatal unit of a University Hospital from 2018 to 2020. During the study period, we approached all mothers whose pregnancies were complicated by diabetes (IDM group) and mothers whose pregnancies were not complicated by diabetes (non-IDM group), for a potential enrollment; we consecutively enrolled those mothers who agreed to participate, including maternal-infant pairs of the IDM group, and maternal-infant pairs of the non-IDM group, matched for gestation (± one week) and birth weight (± 100 g) in a 2:1 ratio (**Figure 1**). Maternal diabetes was diagnosed according to the guidelines of the American Diabetes Association, including pre-existing diabetes and gestational diabetes mellitus (12). The study was approved by the institutional ethics committee (29285/20-06-2018), whereas informed parental consent was obtained as appropriately.

For every infant, we recorded the perinatal characteristics, including gestational age, birth weight, gender, small-for-gestational-age status, delivery mode, maternal preeclampsia, and maternal anthropometrics, including weight, height, and body mass index (BMI) at the beginning and the end of the pregnancy. Prematurity was defined as any pregnancy delivered before 37 weeks of gestation. Maternal weight and height were measured at the first prenatal visit and birth. BMI was calculated by the formula *BMI = weight/height^2^*, while a BMI ≥ 30 kg/m^2^ was utilized to define obesity (13).

### Blood sampling and analyses

Erythrocyte parameters were measured on routine blood analysis for every maternal-infant pair. A peripheral arterial blood sample was collected from each infant within the first twelve hours after birth and erythrocyte parameters [hemoglobin, hematocrit, reticulocytes, MCHr, MCV, percent (%) hypochromia, ferritin] were analyzed with an automated hematology analyzer (Alinity Hq, Abbott, Abbott Park, Illinois, USA). The percentage of hypochromic red blood cells (%hypochromia) can be derived from the hemoglobin concentration distribution curve and is usually defined as the percentage of red blood cells with a cellular hemoglobin content below 28.0 g/dL. In older children and adults, values above 1% are highly suggestive of iron deficiency (3). C-reactive protein, with a cut-off value of 1.0 mg/dL, was evaluated simultaneously as ferritin to exclude underlying infection. A venous blood sample for evaluating the complete set of the same erythrocyte parameters was also collected for each infant’s mother within the first twelve hours after birth.

Neonatal anemia during the first 24 hours of age was defined by hemoglobin lower than 13 g/dL based on standard criteria (14). Ferritin values below 75 μg/L were considered “low” and above 250 μg/L “high” (15); MCHr values below 29 pg were considered “low” and above 38 pg “high” (11).

### Statistical analysis

Descriptive statistics were calculated for perinatal data and the maternal and neonatal erythrocytic parameters. Continuous variables were expressed as mean ± standard deviation or median (interquartile range), as appropriate. The normality of the distributions of continuous variables was assessed by the Kolmogorov-Smirnov test. Continuous variables were compared using the student’s unpaired t-test or the non-parametric Mann-Whitney test. Categorical variables were expressed as n (%) and compared with the chi-square (x^2^) test or Fisher’s exact test.

The correlation of the neonatal MCHr value (continuous variable) with continuous perinatal characteristics that have been previously reported to affect the neonatal iron status (5, 16–18), including gestation, birth weight, maternal BMI, and the erythrocytic indices (i.e., hemoglobin, hematocrit, MCV, %hypochromia, reticulocytes, ferritin) was examined with Spearman’s rho, with a subgroup analysis between IDMs and non-IDMs. Given the differences in hemopoiesis in preterm compared to term infants, namely low plasma erythropoietin levels, low circulating blood volumes, and insufficient erythropoiesis (18), we also performed a stratified analysis between term and preterm infants. Finally, a linear regression analysis was utilized to evaluate the effect of maternal diabetes on neonatal MCHr, adjusted for factors known to influence neonatal iron status (5, 16–18), namely, maternal obesity at the beginning and the end of the pregnancy, prematurity, small-for-gestational-age status, maternal preeclampsia, and maternal anemia (categorical variables). Amongst the above perinatal factors, only those with a significant effect in univariate analysis, with a p-value cut-off value <0.05, were included in the multivariate model. To examine the possible heterogeneity of the association between maternal diabetes and neonatal MCHr and conduct sensitivity analyses, we also performed a linear regression analysis to evaluate the effect of maternal diabetes on neonatal MCHr, adjusted for factors known to influence neonatal iron status as previously reported, in the subgroups of mothers with preexisting diabetes and mothers with gestational diabetes.

The discordance between neonatal MCHr and neonatal ferritin was evaluated with Kendall’s Coefficient of Concordance W test. Discordance was defined when one metric (MCHr or ferritin) exceeded the high value of reference intervals for infants, and the other fell below the low reference interval (11, 15).

All tests were two-sided, and a p-value less than 0.05 was considered statistically significant (alpha 0.05). A power analysis revealed that a sample size of 126 infants in the IDM group and 74 non-IDM infants would be sufficient to detect a difference of at least 5% in the erythrocytic parameters between groups (based on the mean SD of the first ten measurements), with a power of 0.8 and a type-I error of 0.05. The data were analyzed using SPSS Statistics Version 25.0 (IBM, Chicago, Illinois, USA).

## Results

We initially enrolled 222 maternal-infant pairs. Of them, 22 subjects retrieved consent, therefore, 200 maternal-infant pairs were included in the analysis: 126 IDMs, and 74 non-IDMs (**Figure 1**). The perinatal characteristics of the two groups are depicted in **Table 1**. Of the total, 58 (29%) infants were born preterm, 34 (27%) of the IDM, and 24 (32%) of the non-IDM group. Maternal weight gain and BMI increase during pregnancy were significantly higher in non-IDM compared with the IDM group (12 kg compared to 9 kg, p<0.001, and 4.1 kg/cm^2^ compared to 3.1 kg/cm^2^, p<0.001, respectively). No differences were recorded between the IDM and the non-IDM group in the rate of maternal obesity either at the beginning or the end of the pregnancy (**Table 1**).

**Table 1:** Perinatal characteristics and hematological indices of the study population.

	IDM group (n = 126)	Non-IDM group (n = 74)	p
Gestational age, weeks	37.0 ± 2.5	37.1 ± 2.8	0.727
Preterm neonates	34 (27%)	24 (32%)	0.520
Birth weight, g	2923 ± 715	2803 ± 709	0.253
Gender, male	63 (50%)	28 (37%)	0.080
Small-for-gestational-age status	18 (14%)	14 (19%)	0.430
Preeclampsia	10 (8%)	4 (5%)	0.572
Delivery mode, cesarean section	91 (73%)	49 (65%)	0.339
Maternal weight (beginning of pregnancy), kg	71.9 ± 16.9	67.7 ± 16.1	0.116
Maternal weight (end of pregnancy), kg	79.6 ± 16.1	79.5 ± 15.8	0.962
Maternal weight difference, kg	9 (4-13)§	12 (8-15)§	0.001†
Maternal BMI (beginning of pregnancy), kg/cm^2^	26.4 ± 6.1	24.9 ± 6.7	0.147
Maternal obesity (beginning of pregnancy)	21 (16%)	10 (14%)	0.539
Maternal BMI (end of pregnancy), kg/cm^2^	29.3 ± 5.7	29.3 ± 6.3	0.992
Maternal obesity (end of pregnancy)	42 (33%)	22 (30%)	0.412
Maternal BMI difference, kg/cm^2^	3.1 (1.4-4.6)§	4.1 (2.9-5.6)§	0.001†
Maternal anemia (Hb <11 g/dL)	33 (31%)	14 (22%)	0.217
Maternal ferritin, μg/L	39 (17-75)§	35 (18-66)§	0.676
Maternal CRP, mg/dL	0.70 (0.47-0.96)§	0.60 (0.45-0.86)§	0.333
Maternal hematocrit, %	34.5 ± 3.6	35.4 ± 3.6	0.162
Maternal hemoglobin, g/dL	11.6 ± 1.4	12.1 ± 1.3	0.030†
Maternal MCV, fL	101.4 ± 7.2	100.2 ± 9.6	0.392
Maternal hypochromia, %	4 (2-8)§	4 (2-8)§	0.831
Maternal MCHr, pg	29.8 ± 3.0	29.9 ± 3.3	0.957
Maternal reticulocytes, %	2.1 ± 0.8	2.3 ± 1.3	0.175
Maternal reticulocytes, absolute, cells/μL	1.6 ± 0.6	1.8 ± 1.0	0.114
			
Neonatal anemia (hemoglobin <13 g/dL)	4 (3%)	4 (6%)	0.472
Neonatal ferritin μg/L	199 (127-309)§	171 (114-278)§	0.346
Neonatal CRP, mg/dL	0.18 (0.11-0.29)§	0.20 (0.13-0.31)§	0.437
Neonatal hematocrit, %	49.6 ± 6.2	50.4 ± 7.4	0.473
Neonatal hemoglobin, g/dL	16.4 ± 1.8	16.8 ± 2.2	0.137
Neonatal MCV, fL	121.3 ± 9.4	124.6 ± 10.0	0.049†
Neonatal hypochromia, %	12 (7-18)§	9 (6-16)§	0.117
Neonatal MCHr, pg	32.6 ± 3.6	34.2 ± 2.8	0.003†
Neonatal reticulocytes, %	4.4 ± 1.3	4.4 ± 1.4	0.925
Neonatal reticulocytes, absolute, cells/μL	4.8 ± 1.4	4.9 ± 1.6	0.872

Continuous variables are expressed as mean ± SD or median (IQR). P-values of student’s t-test or Mann–Whitney test. Categorical variables are expressed as n (%). P-values of chi-square test or Fisher’s exact test.

†, statistically significant.

§, non-parametric variables.

IDM, infant of diabetic mother; BMI, body mass index; CRP, C-reactive protein; MCV, mean corpuscular volume; MCHr, mean reticulocyte hemoglobin content.

Most maternal red cell indices of the IDM group were not different compared to the non-diabetic mothers; maternal hemoglobin in the IDM group was significantly lower compared with non-diabetic mothers (**Table 1**). Regarding the neonatal red cell indices, the IDM group had a significantly lower neonatal MCV compared with the non-IDM group (121.3 ± 9.4 fL vs. 124.6 ± 10 fL, p=0.049). Also, the average MCHr of infants in the IDM was significantly lower compared to the non-IDM group (32.6 pg vs. 34.2 pg, p=0.003) (**Table 1**).

Neonatal MCHr was significantly correlated with maternal hypochromia (r=-0.237, p=0.004) and neonatal MCV (r=0.674, p<0.001) (**Table 2**). Within the non-IDM group, neonatal MCHr was not correlated with maternal hypochromia but was significantly correlated with maternal BMI change (-0.475, p<0.001) (**Table 2**). In the stratified analysis, in term infants, MCHr was significantly correlated with maternal ferritin (r=0.206, p=0.022), in addition to maternal hypochromia (r=-0.202, p<0.039), and neonatal MCV (r=0.788, p<0.001) (**Table 2**). In preterm infants, MCHr was significantly correlated with maternal hemoglobin (r=0.313, p=0.046), maternal hypochromia (r=-0.311, 0.048), maternal reticulocytes (0.330, p=0.046), neonatal MCV (0.402, p=0.006) and neonatal hypochromia (r=-0.312, p=0.044) (**Table 2**).

**Table 2:** Correlation between neonatal MCHr and continuous variables of interest in the total cohort, in IDM in comparison to non-IDM infants, and in term in comparison to preterm infants.

	Total cohort		IDMs versus non-IDMs				Term versus preterm infants			
			IDM group		Non-IDM group		Term infants		Preterm infants	
	rho	p	rho	p	rho	p	rho	p	rho	p
**Neonatal MCHr**										
Gestational age	0.020	0.791	-0.002	0.987	-0.006	0.960	-0.072	0.428	0.002	0.990
Birth weight	0.019	0.800	-0.025	0.795	0.107	0.395	-0.043	0.637	-0.041	0.768
Maternal BMI (beginning of pregnancy)	-0.075	0.370	-0.135	0.202	0.076	0.583	-0.058	0.556	-0.084	0.615
Maternal BMI (end of pregnancy)	-0.117	0.161	-0.135	0.203	-0.101	0.465	-0.100	0.305	-0.140	0.401
Maternal BMI difference	-0.130	0.120	-0.025	0.814	-0.475	<0.001†	-0.165	0.089	-0.026	0.879
Maternal ferritin	0.104	0.171	0.189	0.057	-0.048	0.707	0.206	0.022†	-0.076	0.591
Maternal hematocrit	-0.122	0.134	-0.111	0.280	-0.173	0.197	-0.160	0.090	0.011	0.949
Maternal hemoglobin	0.002	0.983	-0.061	0.550	0.046	0.734	-0.133	0.234	0.313	0.046†
Maternal MCV	0.019	0.813	-0.026	0.801	0.130	0.365	0.007	0.944	0.073	0.643
Maternal hypochromia	-0.237	0.004†	-0.269	0.008†	-0.196	0.169	-0.202	0.039†	-0.311	0.048†
Maternal MCHr	0.147	0.112	0.083	0.476	0.268	0.083	0.146	0.170	0.150	0.439
Maternal reticulocytes	0.135	0.110	0.120	0.267	0.131	0.344	0.061	0.537	0.330	0.046†
Neonatal ferritin	-0.035	0.638	-0.029	0.756	-0.089	0.480	-0.046	0.613	-0.013	0.926
Neonatal hematocrit	-0.016	0.839	0.001	0.993	-0.085	0.513	-0.050	0.586	0.091	0.522
Neonatal hemoglobin	0.003	0.972	-0.068	0.473	0.046	0.714	0.023	0.802	-0.057	0.532
Neonatal MCV	0.674	<0.001†	0.648	<0.001†	0.706	<0.001†	0.788	<0.001†	0.402	0.006†
Neonatal hypochromia	-0.078	0.349	-0.103	0.320	0.022	0.878	0.029	0.767	-0.312	0.044†
Neonatal reticulocytes	0.136	0.075	0.072	0.450	0.212	0.102	0.166	0.069	0.158	0.264

Spearman’s rho coefficient.

†, statistically significant.

IDM, infant of diabetic mother; MCHr, mean reticulocyte hemoglobin content; BMI, body mass index; MCV, mean corpuscular volume.

In regression analysis, neonatal MCHr was significantly associated with maternal diabetes [standardized coefficients 0.21, 95% confidence interval (CI) 0.05-0.58, p=0.003] and maternal preeclampsia (standardized coefficients 0.17, 95% CI 0.02-0.92, p=0.019), after adjusting for maternal BMI difference, maternal obesity at the beginning and the end of the pregnancy, prematurity, small-for-gestational-age status, and maternal anemia (**Table 3**). Those results were consistent also when analyzing maternal-infant pairs with pre-existing diabetes [maternal pre-existing diabetes (standardized coefficients 0.27, 95% CI 0.20-0.71, p=0.016) and maternal preeclampsia (standardized coefficients 0.28, 95% CI 0.12-0.98, p=0.047)], and maternal-infant pairs with gestational diabetes [maternal gestational diabetes (standardized coefficients 0.20, 95% CI 0.05-0.62, p=0.007) and maternal preeclampsia (standardized coefficients 0.19, 95% CI 0.10-0.79, p=0.015)], as depicted in **Supplemental Tables 1**, **2**.

**Table 3:** Linear regression analysis (univariate and multivariate) of the association of neonatal MCHr with categorical factors of interest.

	Standardized Coefficients	95% CI	p
**Univariate**			
Neonatal MCHr			
Maternal diabetes	0.22	0.06-0.61	0.003†
Maternal obesity (beginning of pregnancy)	0.95	0.44-2.36	0.180
Maternal obesity (end of pregnancy)	0.75	0.36-1.87	0.183
Prematurity	0.42	0.02-1.42	0.566
Small-for-gestational-age status	0.40	0.18-1.88	0.598
Preeclampsia	0.18	0.06-0.90	0.015†
Maternal anemia	0.17	0.07-2.03	0.837
**Multivariate**			
Neonatal MCHr			
Maternal diabetes	0.21	0.05-0.58	0.003†
Preeclampsia	0.17	0.02-0.92	0.019†

MCHr, mean reticulocyte hemoglobin content; CI, confidence intervals; BMI, body mass index.

Amongst the perinatal factors, only those with a significant effect in univariate analysis, with a p-value cut-off value <0.05, were included in the multivariate model.

†, statistically significant.

A discordance was recorded between neonatal MCHr and neonatal ferritin in 12/200 (6%) infants that were statistically significant when evaluated in the whole study group (Kendall’s W=0.155, p=0.001) (**Table 4A**). Of note, when a stratified analysis was performed in the IDM and the non-IDM group, a discordance was recorded in the IDM group that did not reach statistical significance (10/126 infants, 8%, Kendall’s W=0.413, p=0.064) (**Table 4B**). Finally, between term and preterm infants, the discordance remained significant only in term infants (9/152 infants, 6%, Kendall’s W=0.257, p=0.001) (**Table 4C**).

**Table 4:** Discordance between neonatal MCHr and ferritin, with a subgroup analysis in IDM group and non-IDM group, and term and preterm infants.

**A. Total cohort**					
MCHr, pg					
		Low (<29)	Normal (29–38)	High (>38)	p
Ferritin, μg/L	Low (<75)	5	11	4	0.001†
	Normal (75–250)	8	79	5	
	High (>250)	8	54	5	
**B. IDM group**					
MCHr, pg					
		Low (<29)	Normal (29–38)	High (>38)	p
Ferritin, μg/L	Low (<75)	5	7	8	0.064
	Normal (75–250)	9	43	38	
	High (>250)	2	2	0	
**Non-IDM group**					
MCHr, pg					
		Low (<29)	Normal (29–38)	High (>38)	p
Ferritin, μg/L	Low (<75)	0	2	2	0.106
	Normal (75–250)	1	36	3	
	High (>250)	0	16	5	
**C. Term infants**					
MCHr, pg					
		Low (<29)	Normal (29–38)	High (>38)	p
Ferritin, μg/L	Low (<75)	2	3	3	0.001†
	Normal (75–250)	5	55	3	
	High (>250)	6	43	5	
**Preterm infants**					
MCHr, pg					
		Low (<29)	Normal (29–38)	High (>38)	p
Ferritin, μg/L	Low (<75)	3	8	1	0.442
	Normal (75–250)	3	24	2	
	High (>250)	2	11	0	

Categorical variables are expressed as n (%). P-values of Kendall’s Coefficient of Concordance W test.

†, statistically significant.

MCHr, mean reticulocyte hemoglobin content; IDM, infant of diabetic mother.

## Discussion

In a cohort of 200 infants, we found significantly lower neonatal MCHr and MCV in IDMs compared to non-IDMs. Even though there were significantly more mothers with anemia in the IDM compared to the non-IDM group, neonatal MCHr was significantly associated with maternal diabetes and preeclampsia after adjusting for maternal anemia, maternal obesity, prematurity, and small-for-gestational-age status. Neonatal MCHr during the first postnatal day was significantly correlated with maternal hypochromia and neonatal MCV. Among infants from uncomplicated pregnancies, neonatal MCHr was also strongly correlated with maternal BMI change during pregnancy. Finally, a significant discordance was noted between neonatal MCHr and neonatal ferritin, specifically among full-term infants, whereas the discordance between these two hematologic indices in IDMs was not statistically significant.

Our findings are in line with current evidence suggesting that during the first postnatal day, IDMs have evidence of iron deficiency based on hematological indices, specifically MCV and MCHr. Moreover, maternal diabetes and preeclampsia were independently associated with reduced neonatal MCHr in our cohort. Maternal diabetes is associated with depleted fetal iron stores (4–6, 19). The level of maternal glycaemic control and the associated vasculopathy are thought to lead to chronic intrauterine hypoxia and increased erythropoiesis with resultant polycythemia and increased iron demands for the fetus (6). This increased iron demand may exceed placental iron transport capacity because the transferrin binding capacity in the placentae of diabetic mothers is reduced (6), and placental vascular disease further limits iron transport across the placenta (17). Maternal preeclampsia has also been associated with placental vasculopathy and a further decrease in the transplacental iron transport.

Previous studies suggested that infants born to diabetic mothers synthesized significantly higher levels of fetal hemoglobin (20), and authors have found a significant delay in the switch from δ-globin to β-globin in full-term infants born to diabetic mothers, compared with the infants of non-diabetic mothers (21). Furthermore, MCHr has been found significantly lower in cases of a reduced β-globin synthesis, such as beta thalassemia trait, compared to healthy controls (22, 23). The above mechanism, although our study could not test this hypothesis, might also explain the decreased MCHr levels that were recorded in IDMs.

In infants, several clinically used iron status parameters such as ferritin, soluble transferrin receptor, transferrin saturation, and zinc protoporphyrin were poor predictors of iron deficiency (24–26). On the other hand, several studies in children have suggested that MCHr, could be a significant predictor of iron deficiency before the manifestation of anemia (9, 10, 27–31). The neonatal MCHr mean values in the present study were between 32.6 ± 3.6 pg to 34.2 ± 2.8 pg and these are comparable to prior large cohort studies in which MCHr values in term and preterm infants were between 30.3 ± 1.1 pg (27) to 35.9 ± 3.1 pg (11, 26, 29, 32). An MCHr cutoff threshold of 26.9 pg (10) to 28.1 pg (26) has been proposed to indicate iron deficiency and predict anemia. However, since anemia is a late sign of iron deficiency, the optimal MCHr threshold for predicting iron deficiency in infants may be higher, especially during the first week of age (10). Given that iron is essential to neural myelination and neurotransmitter function, playing an important role in the development of the central nervous system (1, 7, 14, 33, 34), the early detection of iron deficiency would be crucial in high-risk infants (34).

In the present study, neonatal MCHr was strongly correlated with maternal hypochromia and neonatal MCV. When analyzed separately, in the preterm cohort, we found a strong correlation of neonatal MCHr with maternal hemoglobin, hypochromia, and reticulocytes. Hypochromic maternal erythrocytes reflect deranged maternal iron status. It is, therefore, not surprising that this finding was associated with a lower neonatal MCHr and MCV, likely due to decreased transplacental iron transport. In line with our findings, other studies have reported a strong independent association between MCHr and erythrocyte indices in infants (10, 29, 32). An interesting finding of the present study is that, although neonatal MCHr was not associated with BMI or maternal obesity either at the beginning or the end of the pregnancy, it was strongly correlated with maternal BMI change during pregnancy among infants of the group of uncomplicated pregnancies. Potential explanations for this novel finding include the expansion of maternal blood volume and a substantial increase in iron demand (35) in the setting of excessive maternal weight gain during pregnancy. In addition, overweight status has been associated with an elevation of hepcidin and serum ferritin and, thus, a decreased iron absorption from the diet (16).

Our findings do not support any strong correlation between neonatal MCHr and neonatal ferritin. On the contrary, a significant discordance was noted between neonatal MCHr and neonatal ferritin levels. As previously noted, this discordance was significant among term infants regardless of maternal diabetes status. In line with our findings, Bahr et al., who evaluated 190 paired ferritin and MCHr measurements, reported a marked discordance in 8% of the samples in a neonatal population (3). When ferritin and MCHr were discordant, other erythrocytic indices, such as erythrocyte microcytosis and hypochromasia, suggested that MCHr gave a more accurate interpretation of iron status (3). Along the same line, German et al. examined the trends of MCHr and ferritin in critically ill infants and concluded that there was a poor correlation between them (26). Factors that influence neonatal ferritin concentration at birth include duration of gestation, fetal sex, or nutrition (36). Furthermore, serum ferritin concentrations are also elevated during periods of infection, or inflammation, when serum ferritin behaves as an acute-phase reactant (37). The pathogenesis of hyperferritinemia is thought to be cytokine-mediated, with interleukin (IL)1a, IL1b, IL6, IL18, tumor necrosis factor-a, c-interferon, and macrophage-colony stimulating factor all implicated. Other inflammatory infectious conditions also produce elevations in ferritin, usually with elevated levels of C-reactive protein (37). In our study, as per study design, we excluded subjects with elevated CRP, however, the impact of the other non-infectious factors on neonatal ferritin levels could not be excluded. In contrast, MCHr reflects the current iron availability for hemoglobin synthesis due to the very short lifespan of reticulocytes (1–2 days in circulation) compared to erythrocytes (life span of 120 days) and is not influenced by inflammation (38, 39). Following an erythropoietic stimulus, iron begins to be incorporated into developing red cells with the appearance of reticulocytes at 48–96 h (38, 39). Besides, in infants with low ferritin but normal MCHr, it might be indicated that normal erythropoiesis is maintained at the expense of diminishing iron stores (3). Thus, MCHr concentrations may be more reflective of changes in iron bioavailability (34). In summary, our findings support that in high-risk infants, such as infants of mothers whose pregnancy was complicated by diabetes, MCHr could be a useful biomarker of iron bioavailability which has implications for hematopoiesis and neurodevelopment.

The findings of the present study should be interpreted in the context of certain limitations. We acknowledge that this was a single-center study, and the population was relatively homogeneous (predominantly Caucasian). Thus, the results may not be generalizable in other populations, especially in resource-poor settings, in racially/ethnically diverse populations, and in the setting of concurrent additional nutritional deficiencies. Furthermore, our sample size was relatively small, while we could not exclude a possible selection bias; however, we included a significant proportion of the available for enrolment maternal-infant pairs of the IDM group during the study duration, whereas the power analysis suggested that our study sample was sufficient to detect a difference of at least 5% in the erythrocytic parameters between groups. We did not measure other markers of iron status, such as serum transferrin or zinc protoporphyrin; therefore, the evaluation of the correlation between neonatal MCHr and other erythrocytic indices and ferritin was limited. Moreover, since we measured the erythrocyte parameters only within the first twelve hours after birth, we could not evaluate whether the lower neonatal MCHr values of IDMs compared to non-IDMs were transient or long-term. Previous studies have reported that MCHr values normally drop over the first few days, while factors such as prematurity or the stress of delivery might be implicated in a higher fall (11, 26, 27, 29). As per our study design, we could not evaluate whether MCHr dropped during the first postnatal days or the predictive value of neonatal MCHr for the later development of anemia or impaired neurodevelopment. Further regular monitoring of the erythrocyte indices would be warranted within the first months of life, to examine whether IDMs develop iron-deficient anemia at a higher rate compared to controls.

In conclusion, neonatal MCHr was significantly lower in IDM compared to infants from uncomplicated pregnancies and neonatal MCHr was significantly associated with maternal diabetes and preeclampsia after adjusting for perinatal factors. Neonatal MCHr during the first postnatal day presented a significant discordance with neonatal ferritin. Further studies are warranted to evaluate the predictive value of MCHr in the later development of anemia and impaired neurodevelopment in high-risk infants.

## Data availability statement

The raw data supporting the conclusions of this article will be made available by the authors, without undue reservation.

## Ethics statement

The studies involving human participants were reviewed and approved by the Aristotle University ethics committee (29285/20-06-2018). Written informed consent to participate in this study was provided by the participants’ legal guardian/next of kin.

## Author contributions

EB and VS conceptualized and developed the study design. Data collection was performed by EB, TM, and KD, DR performed the statistical analysis and wrote the initial draft. EB, HC, GM, TM, KD, CT, AK, DG, and VS reviewed and revised the manuscript. All authors contributed to the article and approved the submitted version.

## Conflict of interest

The authors declare that the research was conducted in the absence of any commercial or financial relationships that could be construed as a potential conflict of interest.

## Publisher’s note

All claims expressed in this article are solely those of the authors and do not necessarily represent those of their affiliated organizations, or those of the publisher, the editors and the reviewers. Any product that may be evaluated in this article, or claim that may be made by its manufacturer, is not guaranteed or endorsed by the publisher.
