# Supplementary material for: Maternal diabetes and the role of neonatal reticulocyte hemoglobin content as a biomarker of iron status in the perinatal period
Source: Front Endocrinol (Lausanne). 2022 Nov 8;13:1011897. doi: 10.3389/fendo.2022.1011897 (PMC9679283; doi:10.3389/fendo.2022.1011897)
Supplement: Supplementary file 2 [file Table_1.docx]

**Supplemental Table 1.** Linear regression analysis (univariate and multivariate) of the association of neonatal MCHr with categorical factors of interest including infants of mothers with pre-existing diabetes and the non-IDM group.

|  | **Standardized Coefficients** | **95% CI** | **p** |
| --- | --- | --- | --- |
| **Univariate** |  |  |  |
| Neonatal MCHr |  |  |  |
| Maternal pre-existing diabetes | 0.27 | 0.03-0.66 | 0.013† |
| Maternal obesity (beginning of pregnancy) | 0.13 | 0.05-2.67 | 0.250 |
| Maternal obesity (end of pregnancy) | 0.17 | 0.06-2.60 | 0.143 |
| Prematurity | 0.30 | 0.13-1.76 | 0.784 |
| Small-for-gestational-age status | 0.10 | 0.08-1.82 | 0.926 |
| Preeclampsia | 0.38 | 0.07-0.92 | 0.041† |
| Maternal anemia | 0.09 | 0.06-2.46 | 0.791 |
|  |  |  |  |
| **Multivariate** |  |  |  |
| Neonatal MCHr |  |  |  |
| Maternal pre-existing diabetes | 0.27 | 0.20-0.71 | 0.016† |
| Preeclampsia | 0.28 | 0.12-0.98 | 0.047† |

MCHr, mean reticulocyte hemoglobin content; CI, confidence intervals; BMI, body mass index.

Amongst the perinatal factors, only those with a significant effect in univariate analysis, with a p-value cut-off value <0.05, were included in the multivariate model.

†, statistically significant.
