# Supplementary material for: Maternal diabetes and the role of neonatal reticulocyte hemoglobin content as a biomarker of iron status in the perinatal period
Source: Front Endocrinol (Lausanne). 2022 Nov 8;13:1011897. doi: 10.3389/fendo.2022.1011897 (PMC9679283; doi:10.3389/fendo.2022.1011897)
Supplement: Supplementary file 3 [file Table_2.docx]

**Supplemental Table 2.** Linear regression analysis (univariate and multivariate) of the association of neonatal MCHr with categorical factors of interest including infants of mothers with gestational diabetes and the non-IDM group.

|  | **Standardized Coefficients** | **95% CI** | **p** |
| --- | --- | --- | --- |
| **Univariate** |  |  |  |
| Neonatal MCHr |  |  |  |
| Maternal gestational diabetes | 0.21 | 0.04-0.56 | 0.006† |
| Maternal obesity (beginning of pregnancy) | 0.13 | 0.09-1.85 | 0.881 |
| Maternal obesity (end of pregnancy) | 0.64 | 0.07-1.71 | 0.468 |
| Prematurity | 0.68 | 0.63-1.62 | 0.387 |
| Small-for-gestational-age status | 0.26 | 0.08-1.35 | 0.746 |
| Preeclampsia | 0.20 | 0.03-0.59 | 0.010† |
| Maternal anemia | 0.15 | 0.09-1.76 | 0.523 |
|  |  |  |  |
| **Multivariate** |  |  |  |
| Neonatal MCHr |  |  |  |
| Maternal gestational diabetes | 0.20 | 0.05-0.62 | 0.007† |
| Preeclampsia | 0.19 | 0.10-0.79 | 0.015† |

MCHr, mean reticulocyte hemoglobin content; CI, confidence intervals; BMI, body mass index.

Amongst the perinatal factors, only those with a significant effect in univariate analysis, with a p-value cut-off value <0.05, were included in the multivariate model.

†, statistically significant.
